# Predictors of change in early child development among children with stunting: Secondary analysis of a randomized trial in Uganda

**DOI:** 10.1371/journal.pgph.0003456

**Published:** 2024-08-15

**Authors:** Joseph Mbabazi, Hannah Pesu, Rolland Mutumba, Gareth McCray, Christian Ritz, Suzanne Filteau, André Briend, Ezekiel Mupere, Benedikte Grenov, Henrik Friis, Mette F. Olsen

**Affiliations:** 1 Department of Nutrition, Exercise and Sports, University of Copenhagen, Copenhagen, Denmark; 2 Department of Paediatrics and Child Health, Makerere University, Kampala, Uganda; 3 School of Medicine, Keele University, Keele, Staffordshire, United Kingdom; 4 The National Institute of Public Health, University of Southern Denmark, Odense, Denmark; 5 Faculty of Epidemiology and Population Health, London School of Hygiene and Tropical Medicine, London, United Kingdom; 6 Faculty of Medicine and Health Technology, Tampere Center for Child, Adolescent and Maternal Health Research, Tampere University and Tampere University Hospital, Tampere, Finland; 7 Department of Infectious Diseases, Rigshospitalet, Copenhagen, Denmark; University of Washington, UNITED STATES OF AMERICA

## Abstract

Millions of children under 5 years in low- and middle-income countries fail to attain their development potential with accruing short- and long-term consequences. Low length/height for age (stunting) is known to be a key factor, but there is little data on how child characteristics are linked with developmental changes among children with stunting. We assessed the socioeconomic, household, anthropometric, and clinical predictors of change in early child development (ECD) among 1–5-year-old children with stunting. This was a prospective cohort study nested in a randomized trial testing effects of lipid-based nutrient supplementation among children with stunting in Uganda. Development was assessed using the Malawi Development Assessment Tool (MDAT). Multiple linear regression analysis was used to assess for predictors of change. We included 750 children with mean ±SD age of 30.2 ±11.7 months 45% of whom were female. After 12 weeks, total MDAT z-score increased by 0.40 (95%CI: 0.32; 0.48). Moderate vs severe stunting, higher fat-free mass, negative malaria test and no inflammation (serum α-1-acid glycoprotein <1 g/l) at baseline predicted greater increase in ECD scores. Older age and fat mass gain predicted a lesser increase in ECD. Our findings reinforce the link between stunting and development with more severely stunted children having a lesser increase in ECD scores over time. Younger age, freedom from malaria and inflammation, and higher fat-free mass at baseline, as well as less gain of fat mass during follow-up predicted a higher increase in developmental scores in this study. Thus, supporting fat-free mass accretion, focusing on younger children, and infection prevention may improve development among children with stunting.

## Introduction

Over 250 million children under 5 years of age in low- and middle-income country settings (LMICs) are unable to reach their full development potential in physical, cognitive, and socioemotional domains [1]. This has both short- and long-term implications including on their educational attainment and executive functioning in adult life which, in turn, affects productivity and consequently propagates the poverty cycle [2]. Notable risk factors include, poor home stimulation, child stunting, and micronutrient deficiencies especially in iron and iodine [3]. With the prevailing conditions including likely global economic recessions, climate change, and the post-corona pandemic context, the number of affected children is likely to increase [4]. Various stakeholders have thus called for interventions integrating health, nutrition, and stimulation to promote early child development (ECD). However, there is limited evidence on feasible approaches to alleviate delayed child development.

Child stunting, defined by a height-for-age z-score (HAZ) below -2 [5], is a form of chronic undernutrition, and has been associated with poor ECD [6]. Stunting is the most prevalent form of undernutrition with over 149 million children under 5 years affected globally [7] and is a key target for reduction (at least by 40%) by 2025 [8]. Since children tend to acquire skills by interacting with people and their surroundings [9], malnourished children including those with stunting and/or micronutrient deficiencies are more likely to be disadvantaged as they tend to be passive with limited exploration of their environment [10,11] compared to children without stunting. Furthermore, nutrient deficiencies are likely to impair brain development and cognition [12]. Factors associated with development among children with stunting are not well documented, although among well-nourished children, socioeconomic status, father’s involvement in child rearing, head circumference, and home stimulation have been associated with improved ECD [13,14].

We previously conducted a randomized community-based 2x2 factorial trial where we found no effect of milk protein (MP) vs soy, or whey permeate (WP) vs maltodextrin in large-quantity lipid nutrient supplement (LNS), or of LNS vs no LNS on ECD among 1–5-year-old stunted Ugandan children [15]. Stunting has been associated with poor cognitive development although the causal relationship is unknown [16]. We sought to assess the factors associated with change in ECD over the 12-weeks’ follow-up period. Our findings may inform ECD programs and other relevant key stakeholders about methods to alleviate developmental delay among stunted children and its likely consequences.

## Materials and methods

### Study design

We conducted a prospective cohort study using data collected in stunted children enrolled as part of the MAGNUS trial (ISRCTN13093195) from 7^th^ February to 17^th^ September 2020. Village-level screening was temporarily halted between 26^th^ March to 14^th^ June due to global corona virus pandemic. The study protocol [17] and main trial outcome [18] have already been described elsewhere. The current study considered all 750 children with stunting who were enrolled and followed-up regardless of study arm.

### Study participants

The study was conducted in Jinja district in East Central Uganda at two public health facilities of Walukuba division (Jinja city) and Buwenge Town Council (Jinja rural). Study participants were recruited from the surrounding villages with the help of village health teams. Children with HAZ <-2 and weight-for-height z-score (WHZ) ≥-3, were referred to the nearby study clinic for eligibility screening. Those with severe acute malnutrition (SAM) marked by WHZ <-3, mid-upper-arm circumference (MUAC) <115 mm, or bilateral pitting oedema were referred for appropriate treatment.

Children were enrolled at the study clinics if i) they lived within the catchment area, ii) had caregivers willing to return for follow-up visits, and iii) agreed to phone-follow-up plus home visit. Children were excluded if they had i) SAM, ii) medical complications requiring hospitalization, iii) peanut or milk allergy history, iv) obvious disability impeding eating or measurement of anthropometric outcomes, v) were participating in another study, vi) the family planned to relocate in the subsequent six months or, vii) the family had another participating child from the same household.

### Study visits

Participants returned fortnightly to refill supplement (intervention group) or laundry soap (reference group) until the 12^th^ week, as described in detail elsewhere [17,18]. At baseline, data was collected on demography, dietary intake including breastfeeding, food insecurity, household income, and anthropometry. Blood samples were collected for assessment of haemoglobin, malaria infection, and acute-phase proteins. Child development assessment and scoring of the child’s participation level during tests were done at baseline and endline. The child was assessed on how happy, engaged, cooperative, and anxious they were during majority of the tests based on an adapted version of the Behaviour Observation Inventory from the Bayley Scales of Infant and Toddler Development [19] as previously used to support MDAT assessment [20,21]. Additionally, caregivers were asked about the child’s developmental stimulation at home using an African-validated family care indicators (FCI) questionnaire [22] at baseline. These were asked about the home stimulation level in four subscales of (i) availability of any child reading materials, (ii) sources of play materials, (iii) variety of play materials, and (iv) engagement with any adult member (15+ years old) in various interactive activities at home. The interactive activities included reading books, telling stories, singing songs, taking the child out, playing with the child, and counting or drawing for the child. The FCI scale has been validated and adapted for use including in such settings [22,23].

### Outcomes

Change in child development was determined as the difference between the endline and baseline developmental score. Development was measured using the Malawi Development Assessment Tool (MDAT) version 6 [24] translated into Lusoga and Luganda. This tool was developed in an African setting and is adapted and validated for use in LMICs including Uganda. It focuses on four domains gross motor, fine motor, language, and social skills development with 39, 42, 40 and 36 milestones in each domain, respectively. The MDAT is primarily an observation-based tool with standardized items assessed by a trained research assistant referred to as a child development officer (CDO).

During assessments, most of the milestones are observed while some, mainly in the social domain, are assessed based on caregiver report. Normal age-specific reference values for each domain are used as a starting point while testing each child. The CDO first performed a forward test until the child failed six consecutive items thereby marking the rest of the items above as failed. If the child passed six consecutive items in the forward test, all items below were marked passed; otherwise, a backward test was performed until six consecutive items were passed. After every twenty assessments, per CDO, a quality check was performed. Specifically, another CDO performed a concurrent assessment and results were compared. In case of any discrepancy, consensus was arrived at in consultations with the respective standard operating procedure and views from other CDOs. The child and caregiver’s participation during the MDAT assessment was observed by the CDO.

### Potential predictors of change in child development

Questionnaires were used at baseline to collect sociodemographic and socioeconomic data. Breastfeeding status was assessed by asking the mother if the child was still breastfeeding. Dietary intake was based on 24-hour recall. Food security was calculated using the USAID household food insecurity access scale [25] while dietary diversity was calculated based on the WHO global nutrition monitoring framework operational guidance [26]. All caregivers received nutrition counselling using the national guidelines on infant and young child feeding [27]. Household wealth index was determined using a 3-stage principal components analysis as used in the demographic and health survey program [28,29].

Anthropometric measurements were done in triplicate and the median used. Participant’s weight, height/length, and head circumference at baseline were all measured as described elsewhere [18]. Body composition was measured in duplicate by bioelectrical impedance using Bodystat 500 machine at 50 kHz (Bodystat, Isle of Man, United Kingdom) as described in detail in the main trial paper [18] and in the methodology paper [30]. We used both the baseline, and change in body composition level over the follow-up period.

Venous blood was drawn from each child, transported to the field laboratory, processed, and temporarily stored at -20°C before being transported to Kampala for storage at -80°C. Processed samples were later transferred to Germany on dry ice for analysis of the acute phase proteins using sandwich enzyme-linked immunosorbent assay (Vitmin Lab, Willstaett, Germany). Before processing, whole blood was used to diagnose malaria (rapid diagnostic test RDT, SD bioline malaria Ag Pf, Abbott, USA) and to measure haemoglobin concentration (Hb201+, HemoCue, Sweden).

### Data management and statistical analyses

Data were collected using paper case report forms (CRF) and double entered in EpiData (Epidata Association, Odense, Denmark) with inbuilt range checks before periodic submission to a secure server using REDCap (Open-Source Vanderbilt University). Statistical analysis was done using Stata SE14 (StataCorp LP, College Station, TX, USA). Descriptive statistics are reported as mean ±SD, median [interquartile range] and frequency, %(n).

We generated MDAT developmental age-adjusted z-scores via a procedure similar to that used in the recent GSED tool [31] (code available on request). The DAZ values were normed on the study sample rather than on an external sample as the study sample was sufficiently large to allow accurate parameter estimation. The advantage of norming within study is that the Scores and the DAZ values do not rely on an external standard that might not generalize well to the data in this study. To calculate DAZ values, first, an item response theory (IRT) analysis was conducted using unidimensional 2-parameter-logistic (2PL) [32] models in the R package *‘mirt’* [33] to create both overall (total) and domain specific scores for each child at each timepoint. Thereafter, a GAMLSS was utilized, using the R package *‘gamlss’* [34] to generate age contingent z-score measures of ability based upon the development scores from IRT model. See S1 Fig for plots of an example of GAMLSS model fit.

Multiple linear regression analysis was used to assess predictors of change in gross motor, fine motor, language development, social skills and the total MDAT z-scores. Potential predictors included sociodemographic status (age, sex, urban residence, household size, and LNS intervention), and socioeconomic factors (multiple income earners, maternal schooling, female-headed households [FHH], food expenditure, food security, dietary diversity, breastfeeding and wealth index).

Family care indicators, as proxies for household stimulation, were also assessed for their prediction. These included having any children’s book at home, >2 sources of play materials, >3 varieties of play materials, interaction with older family members (15+ years old) in > 3 interactive activities at home, and a combination of all the four as described elsewhere including choice of cutoffs [15,35].

We also assessed anthropometric factors for association with change in development including HAZ, WHZ, and head circumference (mm). Additionally, we assessed prediction by body composition data: fat mass (FM), and fat-free mass (FFM) in kilograms, and fat mass index (FMI) and fat-free mass index (FFMI) in kg/m^2^ which are independent of height. Changes in these variables over the follow-up period were referred to as ΔFM, ΔFFM, ΔFMI, and ΔFFMI.

Clinical factors including malaria (positive RDT), anaemia (Hb <110 g/l), inflammatory markers C-reactive protein (CRP, ≥10 mg/l) and α-1-acid glycoprotein (AGP, ≥1 g/l) were assessed for association with change in development. In all our analysis, we adjusted for age, sex, and intervention with LNS irrespective of dairy. A significance level of 0.05 was applied.

### Ethical statement

The study was approved by the School of Medicine Research and Ethics Committee of Makerere University, Kampala (#REC REF 2019–013) and the Uganda National Council of Science and Technology (SS 4927). A consultative approval was obtained from the Danish National Committee on Biomedical Research Ethics (1906848). The study was conducted in accordance with the principles of the Helsinki Declaration [36] and followed local guidelines for human research. All study staff undertook a course in Good Clinical Practice (GCP) and Human Subject Protection (HSP). Oral and written information was provided in Lusoga, Luganda or English. Before caregivers gave written informed consent, their understanding of the information was checked by a different staff member, using a questionnaire.

## Results

A total of 750 children with stunting aged 12–59 months were enrolled in this study. After 12 weeks, 736 children (98%) completed the follow-up study as reported in detail elsewhere [15,18].

The mean ±SD age was 32.0 ±11.74 months, 45% were female and 55% resided in rural areas (**Table 1**). Only a quarter of the households spent <50% of their income on food and 4.4% had secure food access. A minority (12.7%) of children were currently breastfed (Table 1). Household average combined wealth index score was 0.30 ±1.11. A third of families had at least one children’s book and 31% had more than three varieties of play materials at home, but only 9.5% had a stimulative home environment. The average HAZ at inclusion was -3.02 ±0.74, with a weight of 10.59 ±1.98 kg of which 1.80 ±0.87 kg was fat mass. Around 40% tested positive for malaria while over two-thirds had anaemia.

**Table 1:** Baseline characteristics of 750 children with stunting1.

**Sociodemographic data**		
	Age (months)	32.00 ±11.74
	Girl sex	45.1% (338)
	Urban residence	44.7% (335)
	Household size (≥5 people)	61.1% (458)
**Socioeconomic status and dietary data**		
	Multiple income earners (≥2)	29.8% (223)
	Maternal schooling (yes)	52.6% (375)
	Female-headed households	21.3% (157)
	Income spent on food (<50%)	25.1% (188)
	Food secure	4.4% (33)
	Diverse diet	26.3% (196)
	Currently breastfed	12.7% (95)
	Ate animal-source foods in past 24-hr	58.8% (441)
	Ate plant-source foods in past 24-hr	67.5% (506)
**Wealth quintile (n = 401)**		
	Lowest	20.2% (81)
	Second	20.2% (81)
	Middle	19.7% (79)
	Fourth	20.0% (80)
	Highest	20.0% (80)
**Family care indicator subscales**		
	Have any children’s books at home	33.2% (249)
	Sources of play materials >2	18.9% (142)
	Variety of play materials >3	31.3% (235)
	Family interaction >3	33.6% (252)
	Stimulative home environment^2^	9.5% (30)
**Anthropometric and body composition data**		
	Head circumference (cm)	47.22 ±1.79
	Height-for-age (z-score)	-3.02 ±0.74
	Weight-for-height (z-score)	-0.36 ±0.99
	Fat mass (kg)	1.80 ±0.87
**Clinical factors**		
	Negative malaria rapid diagnostic test	60.4% (445)
	Haemoglobin >110 g/l	35.5% (264)
	Serum C-reactive protein <10 mg/l	78.0% (578)
	Serum α-1-acid glycoprotein <1 g/l	36.4% (270)

^1^Data reported as mean ±SD, median [IQR] and % (n).

For categorical variables, the following references were used: girls vs boys, urban vs rural residence; household size ≥5 vs <5; ≥2 vs ≤1 income earner, yes vs no maternal schooling, income spent on food < vs ≥50%, food secure vs food insecure; diverse diet vs no dietary diversity, currently vs not currently breastfed, ate vs did not eat for each of the respective foods in the previous 24 hours, > vs <2 play material sources, > vs <3 varieties of play materials, and > vs <3 interactions with older family members.

^2^A combination of all the four subscales.

There was significant (p<0.001) change across all MDAT domains at the end of 12-week follow-up. The mean changes in developmental z-scores were: gross motor: 0.37 (95%CI: 0.28; 0.45); fine motor: 0.26 (95%CI: 0.17; 0.35); language: 0.31 (95%CI: 0.22; 0.39); social skills: 0.28 (95%CI: 0.19; 0.36); and total score: 0.40 (95%CI: 0.32; 0.48) (**Table 2**).

**Table 2:** Change in MDAT z-scores among stunted children during the 12 weeks follow up^1^.

Characteristics	Baseline		Endline				
	*n*	Mean ±SD	*n*	Mean ±SD	*n*	Difference (95% CI)	*P* value^2^
Gross motor	750	-0.19 ±1.02	736	0.18 ±0.96	736	0.37 (0.28; 0.45)	<0.001
Fine motor	750	-0.13 ±1.04	736	0.13 ±0.94	736	0.26 (0.17; 0.35)	<0.001
Language	750	-0.15 ±1.03	736	0.16 ±0.94	736	0.31 (0.22; 0.39)	<0.001
Social skills	750	-0.13 ±1.03	736	0.14 ±0.95	736	0.28 (0.19; 0.36)	<0.001
Total score	750	-0.20 ±1.00	736	0.20 ±0.96	736	0.40 (0.32; 0.48)	<0.001

^1^Data are number, mean ±SD, difference (endline-baseline) with 95% CI and *p* value.

^2^*P* value obtained by *t* test.

**NOTE:** These values are for the whole cohort irrespective of intervention with LNS or no LNS as previously presented in the trial papers.

After age, sex, and adjustment for intervention with LNS regardless of milk ingredients, older children had 0.08 (95%CI: 0.01; 0.15) lesser increase in total MDAT z-score (**Table 3A**). This was driven by 0.07 (95%CI: 0.002; 0.14) and 0.08 (95%CI: 0.01; 0.15) lower change in gross motor and language z-scores respectively. Conversely, for each 1 kg fat gain, there was a corresponding 0.23 (95%CI: -0.08; -0.39) lesser increase in the total MDAT z-score and this was driven by lesser increase in all the four MDAT domains (**Table 3B**).

**Table 3:** a. Sociodemographic, socioeconomic and household predictors of change in child development among 736 children with stunting^1^. b. Anthropometric predictors of change in child development among 736 children with stunting. c. Clinical predictors of change in child development among children with stunting^1^.

Characteristics		ΔGross motor		ΔFine motor		ΔLanguage		ΔSocial skills		ΔTotal score	
		β (95% CI)	P	β (95% CI)	P	β (95% CI)	p	β (95% CI)	p	β (95% CI)	p
**Sociodemographic status**											
Age (years)		-0.07 (-0.14; -0.002)	0.04	-0.03 (-0.10; 0.03)	0.33	-0.08 (-0.15; -0.01)	0.02	-0.04 (-0.10; 0.03)	0.29	-0.08 (-0.15; -0.01)	0.02
Girl sex		-0.10 (-0.23; 0.03)	0.14	-0.11 (-0.25; 0.02)	0.10	-0.01 (-0.14; 0.12)	0.90	-0.08 (-0.22; 0.05)	0.23	-0.11 (-0.24; 0.02)	0.11
Urban residence		0.08 (-0.06; 0.21)	0.26	-0.08 (-0.21; 0.06)	0.27	-0.06 (-0.20; 0.07)	0.35	0.002 (-0.13; 0.14)	0.97	-0.04 (-0.17; 0.09)	0.54
Household size (5+ people)		-0.04 (-0.18; 0.10)	0.57	0.06 (-0.08; 0.20)	0.423	0.03 (-0.11; 0.16)	0.70	0.08 (-0.05; 0.22)	0.23	0.02 (-0.11; 0.16)	0.76
LNS (yes)		-0.06 (-0.22; 0.11)	0.50	-0.07 (-0.24; 0.09)	0.40	-0.08 (-0.24; 0.08)	0.32	-0.04 (-0.21; 0.12)	0.60	-0.09 (-0.25; 0.08)	0.30
**Socioeconomic status**											
Multiple income earners (2+)		0.10 (-0.04; 0.25)	0.17	0.11 (-0.03; 0.26)	0.13	0.04 (-0.11; 0.18)	0.63	0.06 (-0.08; 0.21)	0.39	0.07 (-0.07; 0.21)	0.32
Maternal schooling (yes)		0.07 (-0.06; 0.21)	0.29	-0.05 (-0.18; 0.09)	0.47	-0.07 (-0.20; 0.06)	0.30	-0.003 (-0.14; 0.13)	0.97	-0.07 (-0.21; 0.06)	0.27
Female-headed households		-0.04 (-0.20; 0.13)	0.66	0.06 (-0.10; 0.23)	0.46	-0.06 (-0.22; 0.10)	0.46	0.06 (-0.10; 0.22)	0.48	0.01 (-0.15; 0.17)	0.88
Income spent on food (<50%)		-0.07 (-0.22; 0.09)	0.40	-0.04 (-0.19; 0.12)	0.63	-0.05 (-0.20; 0.10)	0.51	0.02 (-0.14; 0.18)	0.80	-0.04 (-0.19; 0.11)	0.58
Food secure [access]		0.21 (-0.11; 0.54)	0.19	-0.04 (-0.36; 0.29)	0.83	0.08 (-0.24; 0.39)	0.64	0.04 (-0.28; 0.36)	0.80	0.08 (-0.24; 0.39)	0.63
Diverse diet		0.08 (-0.08; 0.23)	0.32	0.06 (-0.09; 0.22)	0.43	0.08 (-0.07; 0.23)	0.31	0.10 (-0.05; 0.26)	0.20	0.12 (-0.03; 0.28)	0.11
Currently breastfed		-0.09 (-0.32; 0.14)	0.44	-0.23 (-0.45; -0.002)	0.048	0.02 (-0.20; 0.24)	0.87	-0.03 (-0.25; 0.20)	0.82	-0.06 (-0.29; 0.16)	0.57
Wealth quintile (n = 394)	Lowest	Ref		Ref		Ref		Ref		Ref	
	Second	0.18 (-0.13; 0.48)	0.25	-0.02 (-0.31; 0.26)	0.89	0.07 (-0.20; 0.34)	0.61	0.01 (-0.27; 0.29)	0.95	0.02 (-0.26; 0.31)	0.88
	Middle	0.21 (-0.10; 0.52)	0.19	-0.07 (-0.36; 0.22)	0.63	0.31 (0.03; 0.58)	0.03	0.16 (-0.13; 0.44)	0.27	0.16 (-0.13; 0.45)	0.29
	Fourth	0.07 (-0.23; 0.38)	0.64	-0.23 (-0.51; 0.06)	0.12	-0.03 (-0.30; 0.24)	0.82	0.03 (-0.24; 0.31)	0.81	-0.14 (-0.43; 0.15)	0.34
	Highest	0.12 (-0.18; 0.43)	0.43	0.09 (-0.20; 0.37)	0.54	0.23 (-0.04; 0.50)	0.10	0.14 (-0.14; 0.42)	0.32	0.17 (-0.12; 0.45)	0.24
**Family care indicators**											
Have any children’s book at home		0.11 (-0.03; 0.26)	0.12	-0.005 (-0.15; 0.14)	0.95	0.16 (0.02; 0.30)	0.03	0.13 (-0.01; 0.27)	0.08	0.11 (-0.03; 0.25)	0.11
Sources of play materials >2		0.03 (-0.14; 0.20)	0.76	-0.02 (-0.19; 0.15)	0.84	-0.03 (-0.20; 0.14)	0.74	0.05 (-0.12; 0.22)	0.59	-0.03 (-0.20; 0.13)	0.70
Variety of play materials >3		0.15 (0.001; 0.30)	0.049	0.13 (-0.01; 0.28)	0.08	0.10 (-0.04; 0.25)	0.17	0.09 (-0.06; 0.23)	0.26	0.13 (-0.01; 0.28)	0.08
Family interaction >3		0.09 (-0.05; 0.23)	0.22	0.02 (-0.12; 0.16)	0.79	0.06 (-0.08; 0.20)	0.37	0.08 (-0.06; 0.23)	0.25	0.08 (-0.06; 0.22)	0.27
Stimulative home environment^2^		0.52 (0.14; 0.91)	0.01	0.11 (-0.26; 0.48)	0.55	0.22 (-0.14; 0.59)	0.23	0.39 (0.02; 0.77)	0.04	0.31 (-0.05; 0.67)	0.09

**Characteristics**	**ΔGross motor**		**ΔFine motor**		**ΔLanguage**		**ΔSocial skills**		**ΔTotal score**	
	β (95% CI)	P	β (95% CI)	P	β (95% CI)	p	β (95% CI)	p	β (95% CI)	p
**Baseline values**										
Height-for-age z-score	0.15 (0.05; 0.24)	0.002	0.12 (0.03; 0.21)	0.01	0.16 (0.07; 0.25)	<0.001	0.08 (-0.01; 0.17)	0.07	0.16 (0.07; 0.25)	<0.001
Weight-for-height z-score	0.01 (-0.06; 0.08)	0.84	0.03 (-0.04; 0.09)	0.46	-0.001 (-0.07; 0.07)	0.97	0.01 (-0.06; 0.08)	0.74	0.02 (-0.04; 0.09)	0.50
Head circumference	-0.02 (-0.06; 0.03)	0.55	0.06 (0.01; 0.11)	0.02	0.02 (-0.03; 0.07)	0.39	0.01 (-0.04; 0.06)	0.71	0.03 (-0.02; 0.08)	0.23
Fat mass (kg)	0.04 (-0.04; 0.12)	0.34	0.04 (-0.04; 0.13)	0.29	-0.004 (-0.09; 0.08)	0.91	0.01 (-0.07; 0.10)	0.72	0.04 (-0.04; 0.12)	0.36
Fat-free mass (kg)	0.14 (0.02; 0.27)	0.03	0.10 (-0.02; 0.23)	0.11	0.18 (0.06; 0.30)	0.004	0.07 (-0.05; 0.20)	0.24	0.17 (0.05; 0.29)	0.01
Fat mass index (kg/m^2^)	0.02 (-0.04; 0.08)	0.49	0.03 (-0.03; 0.09)	0.32	-0.003 (-0.06; 0.05)	0.91	0.02 (-0.04; 0.07)	0.60	0.02 (-0.03; 0.08)	0.40
Fat-free mass index (kg/m^2^)	-0.08 (-0.20; 0.04)	0.19	-0.05 (-0.17; 0.07)	0.45	-0.02 (-0.14; 0.09)	0.68	-0.03 (-0.14; 0.09)	0.68	-0.03 (-0.15; 0.09)	0.62
**Change over 12 weeks**										
ΔFat mass (kg)	-0.22 (-0.38; -0.06)	0.01	-0.20 (-0.36; -0.04)	0.01	-0.19 (-0.35; -0.04)	0.02	-0.17 (-0.32; -0.01)	0.04	-0.23 (-0.39; -0.08)	0.003
ΔFat-free mass (kg)	0.31 (0.09; 0.53)	0.01	0.10 (-0.13; 0.32)	0.40	0.09 (-0.13; 0.30)	0.44	0.25 (0.02; 0.47)	0.03	0.16 (-0.06; 0.37)	0.15
ΔFat mass index (kg/m^2^)	-0.16 (-0.26; -0.05)	0.003	-0.12 (-0.23; -0.02)	0.02	-0.11 (-0.21; -0.01)	0.03	-0.12 (-0.22; -0.01)	0.03	-0.15 (-0.25; -0.05)	0.003
ΔFat-free mass index (kg/m^2^)	0.07 (-0.10; 0.25)	0.40	0.01 (-0.16; 0.18)	0.91	0.01 (-0.16; 0.18)	0.94	0.11 (-0.06; 0.28)	0.22	0.02 (-0.15; 0.19)	0.80

**Characteristics**	**ΔGross motor**		**ΔFine motor**		**ΔLanguage**		**ΔSocial skills**		**ΔTotal score**	
	β (95% CI)	P	β (95% CI)	P	β (95% CI)	p	β (95% CI)	p	β (95% CI)	p
**Clinical factors (n = 727)**										
Negative malaria RDT	0.17 (0.03; 0.31)	0.01	0.16 (0.03; 0.30)	0.02	0.26 (0.13; 0.40)	<0.001	0.09 (-0.04; 0.23)	0.18	0.24 (0.11; 0.37)	<0.001
Haemoglobin >110 g/l	-0.01 (-0.15; 0.13)	0.90	-0.05 (-0.19; 0.09)	0.47	0.02 (-0.11; 0.16)	0.73	0.06 (-0.08; 0.20)	0.39	0.03 (-0.11; 0.16)	0.71
Serum CRP <10 mg/l	0.18 (0.01; 0.34)	0.03	0.12 (-0.15; 0.17)	0.88	0.07 (-0.09; 0.23)	0.40	0.07 (-0.09; 0.23)	0.39	0.12 (-0.04; 0.27)	0.15
Serum AGP <1 g/l	0.15 (0.01; 0.29)	0.03	0.18 (0.04; 0.32)	0.01	0.20 (0.06; 0.34)	0.004	0.14 (-0.001; 0.28)	0.052	0.22 (0.09; 0.36)	0.001

^1^Data reported as regression coefficients, β (95% CI) and p value adjusted for age, sex, and intervention with LNS regardless of milk ingredients.

For categorical variables, the following references were used: girls vs boys, urban vs rural residence; household size ≥5 vs <5; 2 or more vs none or single income earner, yes vs no maternal schooling, income spent on food < vs ≥50%, food secure vs food insecure; diverse diet vs no dietary diversity, currently vs not currently breastfed, and ate vs did not eat for each of the respective foods in the preceding 24 hours of the structured interview.

^2^Having any children’s book at home, with >2 sources of play materials of >3 varieties, and with >3 engagements with older family members in interactive activities.

Data reported as regression coefficients, β (95% CI) and p value adjusted for age, sex, and intervention with LNS regardless of milk ingredients.

^1^Data reported as β coefficients with their 95% confidence intervals and p value based on linear regression adjusted for baseline value, age, sex, and intervention with LNS regardless of milk ingredients.

For every unit higher HAZ at baseline, there was a corresponding 0.16 (95%CI: 0.07; 0.25) higher increase in total MDAT z-score (Table 3B). This was as a result of 0.15 (95%CI: 0.05; 0.24), 0.12 (95%CI: 0.03; 0.21), and 0.16 (95%CI: 0.07; 0.25), greater difference in gross motor, fine motor, and language z-scores, respectively. Baseline higher fat-free mass composition was associated with 0.17 (95%CI: 0.05; 0.29) greater increase in total MDAT z-score driven by higher changes in gross motor and language z-scores (Table 3B). Children without malaria or inflammation at baseline had 0.24 (95%CI: 0.11; 0.37) and 0.22 (95%CI: 0.09; 0.36) greater increase in total MDAT z-scores, respectively (**Table 3C**). This was driven by greater changes in gross, fine motor, and language z-scores.

Currently breastfed children at baseline had 0.23 (95%CI: 0.002; 0.45) lesser increase in fine motor z-scores, but no differences from non-breastfed in other domains (**Table 3A**). Children from households with a stimulative home environment were associated with 0.52 (95%CI: 0.14; 0.91) and 0.39 (95%CI: 0.02; 0.77) higher increase in gross motor and social skills z-scores respectively (**Table 3A**). This was partly driven by possession of greater than three varieties of play material and having any children’s books which were associated with 0.15 (95%CI: 0.001; 0.30) higher increase in gross motor and 0.16 (95%CI: 0.02; 0.30) higher increase in language z-scores. Wealth quintile was not associated with changes in development. Each 1 cm higher head circumference at baseline was associated with 0.06 (95%CI: 0.01; 0.11) higher increase in fine motor z-score, but no notable increase in z-scores across other developmental domains (**Table 3B**).

At endline, children were overall more engaged, cooperative and not anxious than at baseline (**S1 Table**).

## Discussion

As in children without stunting, our findings derived from the summative total score reveal that improved development is attainable with higher HAZ scores, at early ages, with fat-free but not fat mass accretion, and being free of infections indicated by inflammation and malaria rapid test. Whereas head circumference was associated with higher increase in fine motor, the reverse was true for prolonged breastfeeding. Coming from a stimulative home was associated with greater increases especially in gross motor and social skill scores.

Our findings on HAZ and head circumference association concur with results from the MAL-ED study [14] from various LMICs. In their study, head circumference was a stronger predictor of ECD at 24 months especially on cognitive development with over ~0.7 cognitive score increases at 24 months per unit z-score increase in head circumference. Conversely, each unit increase in length-for-age z-score resulted in ~0.2 increase in cognitive score [14]. In our study, our point estimates are lower than what was registered in the MAL-ED study that is 0.06 vs 0.7 and 0.12 vs 0.2 for head circumference and HAZ change in fine motor score respectively. Of note, we had much older children (1–5 years vs 0–24 months), who were already stunted followed up for only 3 vs 24 months. The associations between stunting level and head circumference with ECD has already been described elsewhere [6,14], including in our analysis of baseline developmental scores among these children [35].

A study among pre- and full-term children followed up from infancy through preschool age revealed that higher 4-month corrected age to 4-year FFM gain was associated with higher full scale intelligence quotient (IQ) [37]. Among the full-term group, higher 4-month to 4-year fat mass gains were associated with lower full-scale IQ. Although their study was from a high-income setting with a longer follow-up period (48 months), our findings are similar with respect to FFM and fat mass gain. This is also in line with an Ethiopian study where FFM at birth, but not FM, was associated positively with global developmental score (2.48, 95%CI: 0.17; 4.79) at 2 years, mainly attributable to language skills development [38]. There has also been evidence suggesting that lean tissue growth optimizes competing cognitive and metabolic consequences [39]. We are likely to have a similar trend even among stunted children augmented by the fact that, compared to those that were supplemented, unsupplemented children in this study gained mainly fat mass at the expense of FFM [18].

Low acute phase proteins and negative malaria RDT were associated with greater increase in developmental scores, similar to findings from other cohort studies [40]. Healthy children without malaria or inflammation may be more active with lesser toll on their immunity and nutrients hence greater increase in ECD scores. This is also aligned with our baseline findings where those children with elevated serum AGP and positive malaria RDT had lower ECD scores [35].

The early pre- and postnatal period is characterized by rapid maturation of metabolic, endocrine, neural, and immune pathways, which all have a strong bearing on child growth and development [41]. These pathways develop in tandem with a complex assembly program reliant on both internal and external factors which are susceptible to such adversities like infections and suboptimal feeding [41].

Similar to our baseline findings [35], prolonged breastfeeding was associated with lower change but only in fine motor. Contrary to the expected and well documented evidence that breastfeeding is associated with better development [42], it has been noted in recent literature that this relationship is prone to confounding. Further adjustments for socioeconomic status and maternal cognition tend to reduce the association, especially at older ages [43]. Reverse-causality may also contribute because mothers may have preferred to support children with poor anthropometry and/or other complications by prolonging breastfeeding. Indeed, stunted children in this study that were still breastfed had worse anthropometric indices, more cases of anaemia and cobalamin deficiency [35]. Prolonged breastfeeding may be a counter measure for various disruptions which could be partly why we saw lower change in their fine motor scores.

A stimulative home environment marked especially by availability of children’s books, and >3 varieties of play materials was associated with greater increase in gross motor and social skills in agreement with findings from a path analysis of the iLiNs trials [44]. Having books and more varied play materials is likely to promote caregiver activities with children to a greater extent hence more stimulation promoting development. This and other established associations, moreover among impoverished stunted children in Uganda could guide implementation of the ECD policy which is now a focus of full adoption by the government.

Key strengths of our study include a large sample of children with stunting, assessment of a variety of probable predictors, and use of a contextually appropriate tool for assessment of child development (MDAT). Key limitations include inability to establish causal relationships given the observational nature of the reported associations. In addition, we were unable to measure how much change in developmental scores was attributable to the ‘learning effect’ of the MDAT as acknowledged elsewhere [21]. Our finding that children were more cooperative, engaged with enthusiasm, unfearful and not anxious during endline, compared to baseline MDAT assessments suggests that perhaps there was a notable attributable change due to the learning effect of the tool.

## Conclusions and recommendations

Findings from our study reinforce existing ECD management program core objectives such as stunting alleviation to foster good ECD. However, to be effective, these should be timely, focusing on younger children, and should be supported by nutrition interventions supporting fat-free, rather than fat mass accretion. Continued efforts to reduce malaria and inflammation and to promote stimulating home environments may also promote higher ECD changes even among stunted children in LMICs like Uganda.

## Supporting information

S1 Table Mother and Child cooperativeness during MDAT assessments among 736 children with stunting. (XLSX)

S1 Fig Plots of the scaled and Z-scores (DAZ) for total MDAT score. (TIFF)
