# Supplementary figures and images for: Predictors of change in early child development among children with stunting: Secondary analysis of a randomized trial in Uganda
Source: PLOS Glob Public Health. 2024 Aug 15;4(8):e0003456. doi: 10.1371/journal.pgph.0003456 (PMC11326642; doi:10.1371/journal.pgph.0003456)

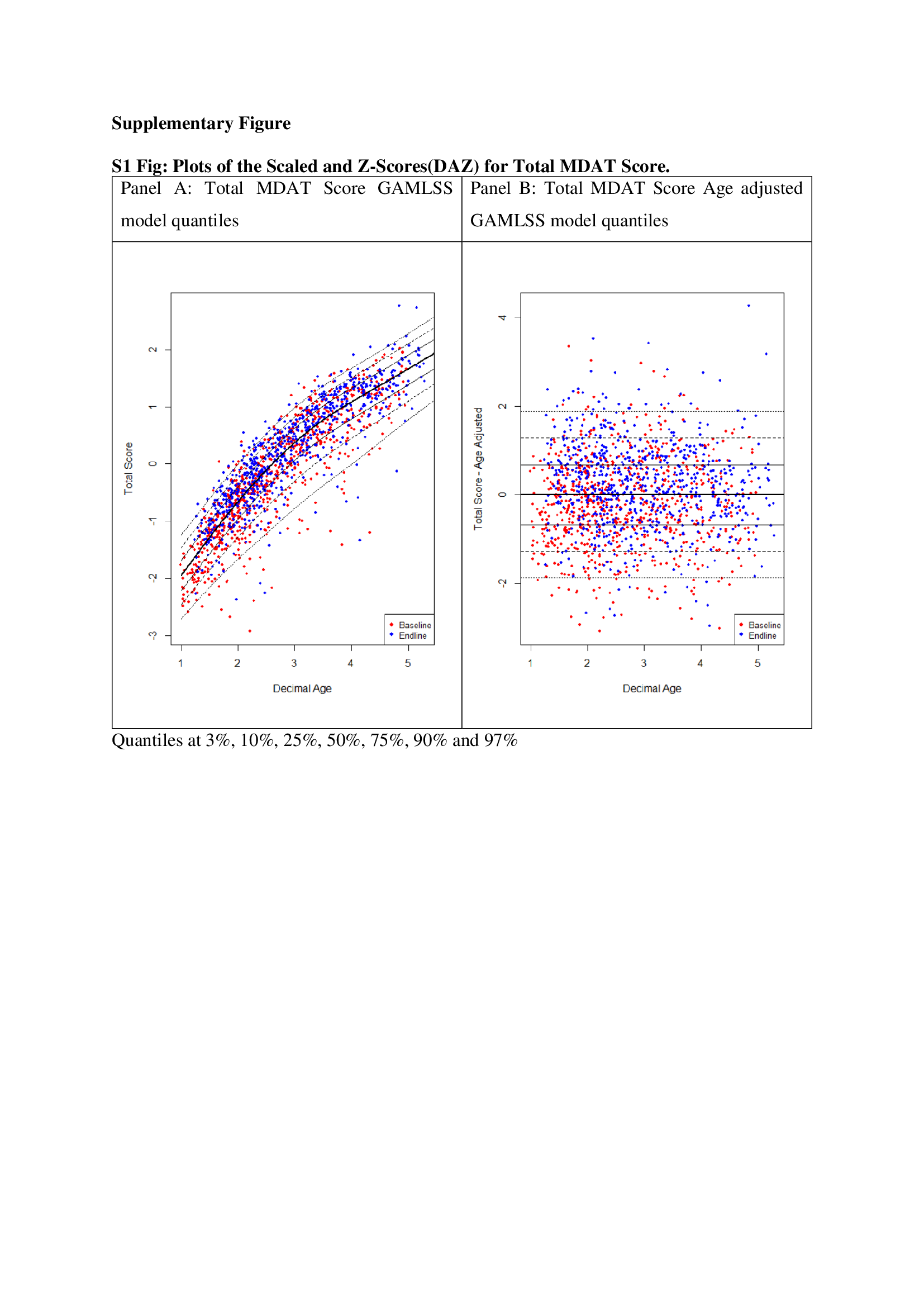

Supplement: S1 Fig — (TIFF) [file pgph.0003456.s002.tiff]
